# Molecular insights into enriched environments and behavioral improvements in autism: a systematic review and meta-analysis

**DOI:** 10.3389/fpsyt.2024.1328240

**Published:** 2024-02-01

**Authors:** Yutong Li, Jing Lu, Jing Zhang, Wenxin Gui, Weijie Xie

**Affiliations:** ^1^ School of Mental Health, Wenzhou Medical University, Wenzhou, China; ^2^ Department of Sports Medicine, The Affiliated Hospital of Qingdao University, Qingdao University, Qingdao, China; ^3^ Clinical Research Center for Mental Disorders, Shanghai Pudong New Area Mental Health Center, Tongji University School of Medicine, Shanghai, China

**Keywords:** enriched environment, autistic spectrum disorder (ASD), neurogenesis, synaptic plasticity, hippocampus, valproic acid (VPA)

## Abstract

**Aims:**

Autism is a multifaceted developmental disorder of the nervous system, that necessitates novel therapeutic approaches beyond traditional medications and psychosomatic therapy, such as appropriate sensory integration training. This systematic mapping review aims to synthesize existing knowledge on enriching environmental interventions as an alternative avenue for improving autism, guiding future research and practice.

**Method:**

A comprehensive search using the terms ASD and Enriched Environment was conducted across PubMed, EMBASE, ISI, Cochrane, and OVID databases. Most of the literature included in this review was derived from animal model experiments, with a particular focus on assessing the effect of EE on autism-like behavior, along with related pathways and molecular mechanisms. Following extensive group discussion and screening, a total of 19 studies were included for analysis.

**Results:**

Enriched environmental interventions exhibited the potential to induce both behavioral and biochemical changes, ameliorating autism-like behaviors in animal models. These improvements were attributed to the targeting of BDNF-related pathways, enhanced neurogenesis, and the regulation of glial inflammation.

**Conclusion:**

This paper underscores the positive impact of enriched environmental interventions on autism through a review of existing literature. The findings contribute to a deeper understanding of the underlying brain mechanisms associated with this intervention.

## 1 Introduction

Autistic spectrum disorder (ASD) is a neurodevelopmental disorder characterized by deficits in social communication. repetitive behaviors, and narrow interests (1). In the United States, approximately 2.3 percent of 8-year-olds and 2.2 percent of adults are affected by autism, with these prevalence rates increasing globally (2). Despite significant advances in autism-related research in recent years, providing a more comprehensive understanding of the disorder, effective treatment for autism remains elusive.

Enriched environments have emerged as a novel therapeutic approach garnering attention for improving outcomes in individuals with autism. An enriched environment refers to a physical and social setting that fosters brain development by providing sensory and cognitive stimulation (3). The concept of an enriched environment (EE) has been applied across various fields, including animal research and human cognitive development. Presently, studies reveal significant protocol variations in EE, including the presence of running wheels, number of cagemates, duration of enrichment, and the age of animals at the onset and conclusion of interventions (4). These specific setting differences complicate comparisons. However, the basic experimental set-up remains the same: animals, typically rats or mice, housed in larger groups within larger cages, often equipped with additional toys, nesting material, and hiding tubes (5). Despite the emphasis on comparison between groups in enrichment environment studies, individual differences are often overlooked.

Numerous studies consistently demonstrate the beneficial impact of EE on behavioral improvements in autism. Moreover, researchers have proposed innovative perspectives at the micro-mechanism level. Environmental enrichment can influence the Nrf/BDNF pathway, promoting neuronal growth, recombination, and recovery, thereby reducing anxiety and enhancing motor abilities (6). The hippocampus, an important region for learning and memory, has been a focal point, with scientists proposing interventions aiming at altering cell excitability and improving synaptic structure and morphology (7–9). Another avenue involves the regulation of neuroinflammation and neuron regeneration syndromes (8–14).

To comprehensively assess the effects of EE on autism, a meta and systematic review was conducted. A subsection of this review focused on the literature exploring the role of environmental enrichment in autism phenotypes, with a primary emphasis on neuroinflammation-related signaling pathways and microglia activity implicated in autism improvement (15). This inquiry aimed to provide insights for future clinical practice and research. In the future, experiments will be carried out to further explore the role of EE in improving the brain microenvironment and preventing clinical diseases.

## 2 Methods

### 2.1 Inclusion and exclusion criteria

#### 2.1.1 Inclusion criteria

There is no limitation on research types. Only articles focusing exclusively on animal studies with autism as the disease phenotype are included, and they must be published in either Chinese or English. The intervention measures in these studies must involve environmental enrichment. The outcome indicators should report improvements in loneliness symptoms, including changes in both behavioral and molecular biological indicators.

(1) There is no limitation on research types; (2) Language learning is limited to Chinese and English; (3) Focusing only on animal studies, the disease phenotype is autism; (4) The intervention measures are environmental enrichment; (5) Outcome indicators should report the improvement of loneliness symptoms, including changes in behavioral and molecular biological indicators.

#### 2.1.2 Exclusion criteria

Excluded are studies that involve combinations of autism with other diseases, studies with missing data or duplicate reports, and articles of theoretical discussions, clinical trials, conference abstracts, reviews, meta-analyses, and other similar types.

(1) Studies involving combinations of autism with other diseases; (2) Studies with missing data or duplicate reports; (3) Theoretical discussions, clinical trials, conference abstracts, reviews, meta-analyses, and other similar article types.

### 2.2 Protocol and registration

A search was conducted on the International Prospective Register of Systematic Reviews (PROSPERO) system under the heading “Autistic Disorder and Environmental Enrichment”. No similar studies were found to be published or registered. While the National Institute for Health Research (NIHR) primarily focuses on outcomes in human health, social care, welfare, public health, and education, this systematic review closely examines results from animal studies. Registration is not currently supported.

### 2.3 Database search

A systematic literature search was performed in electronic databases, including MEDLINE (via PubMed), EMBASE, and ISI Web of Science. Searches were conducted in July 2022, and restricted to animal studies only. Two sets of words were used in the searches: (1) population (ASD animal models) and (2) intervention (enriched environment). Database search strategies are available in **Supplementary Material 1**.

### 2.4 Information extraction

Two evaluators independently screened the literature, extracted data, and cross-checked it. In case of discrepancies, a third party was consulted for judgment. The final decision was made after a group discussion. Data extraction included title, year, study type, modeling method, intervention time and method, behavioral type, and outcome index.

## 3 Results

### 3.1 Literature search and screening

**Figure 1:**
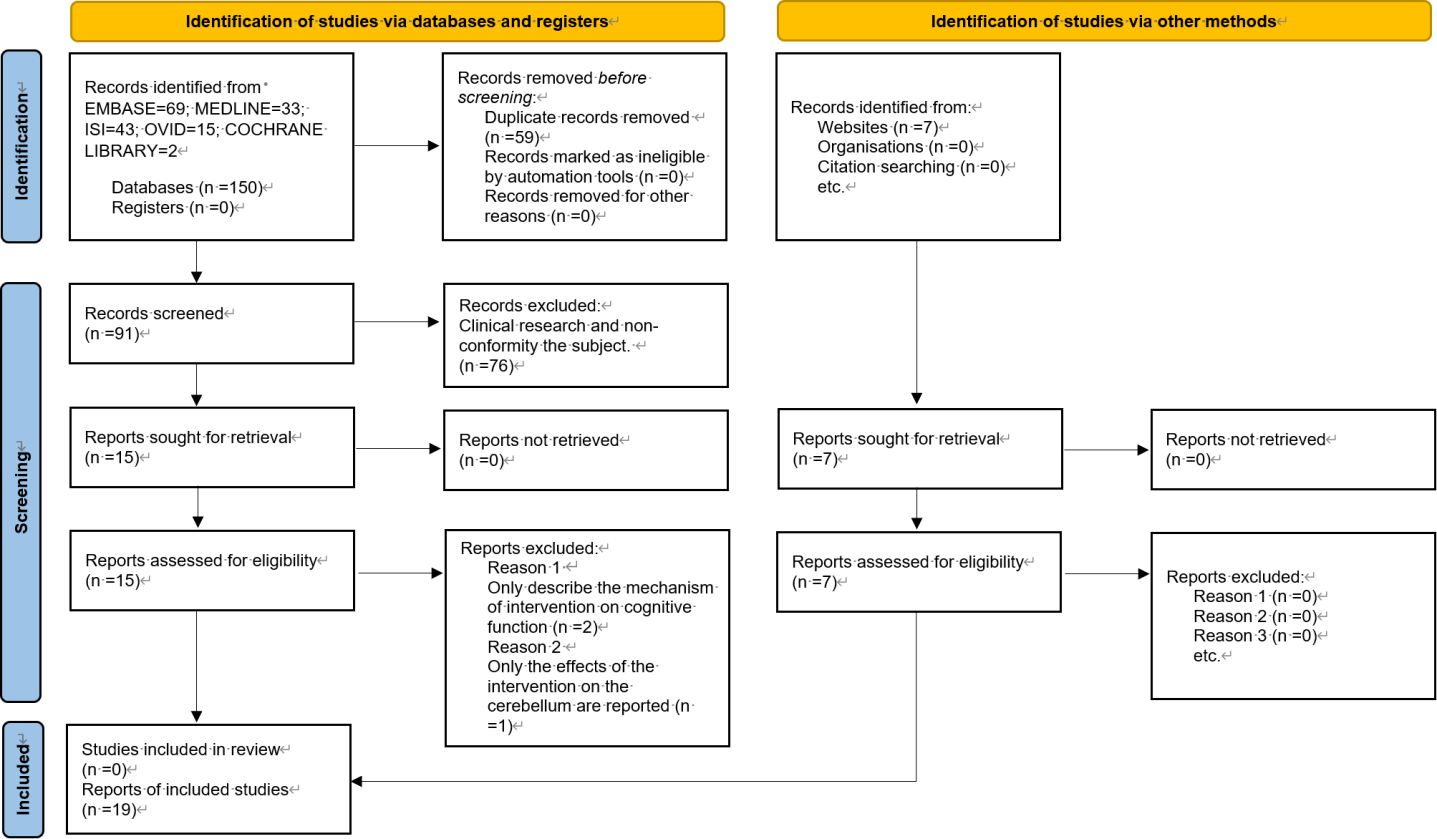
Flow chart of literature screening.

A total of 150 papers were identified, and after the first screening, 91 papers were included. Following the second screening, 19 papers were finally included. The literature screening process and results are illustrated in **Figure 1**.

### 3.2 Basic characteristics and intervention protocols of selected studies

**Table 1** presents the behavioral phenotypic changes induced by the enriched environment, along with information on the intervention period, environment type, and behavioral experiments. To facilitate comparison and avoid interference from modeling approaches, experiments of the same model are listed together. A subset of articles conducted tests on microscopic indicators, such as synaptic morphology, protein expression, and hormone levels. The results of these experiments are separately presented in **Table 2**, offering a perspective for subsequent exploration.

**Table 1:** Basic characteristics of included documents.

Literature	Pattern making	Intervening measure	Intervention weeks	Behavioral experiment	Outcome measurement	PMID
(16)	Exposure to VPA during pregnancy	SE/EE	16	OFT/EPM	↓The levels of anxiety and depression Gender affects the degree of change	33107086
(17)		SE/UE/PE	16	EPM/social test/Y-maze/FC	PE: complete reversal of autism UE: no difference change	26089770
(18)		SE/EE	3	Three-chamber social test	↑ Social skills	
(19)		SE/EE	4.3	Social interaction/texture discrimination/acoustic startle reflex tests	↓ Autism-like cognitive disorders Gender determines the degree of impact	35738908
(20)		SE/EE	1	ALB symptoms (social interaction and repetitive movements)	↑ Cognition, mood and anxiety levels	26089770
(21)		SE/EE/cluster needling	3	OFT/Three-chamber social test	↓ Anxiety and social disorders	
(7)		SE/EE	4	OFT/EPM/NOR/Three-chamber social test	↑ Recognition of memory deficits, anxiety, and social disorders	28655565
(12)		SE/EE	3.4	NOR/von Frey test/EPM、Social interaction, etc.,	↑ Cognitive competence ↓ Autism-like behaviors and anxiety	15920505
(22)	*Mecp2* gene mutation	SE/EE	2	OFT/elevated beam test/EPM/footprint ink test	↑ Motor ability and delayed clasp development ↓ Exploration of the open arm	20634955
(23)		SH/EE	2	Rota-rod/Observe general motor activity	↑ Motor coordination in heterozygous females, but not males	18557922
(24)		SH/EE/RW	7	EPM/NSF/SPT	↑ Emotional stability compared to physical exercise alone	26019053
(25)		SE/EE	1	OFT/Rotor-rod/FC	↑ Motor activity deficits There is no significant change in situational or cue-fear conditioning	18687363
(26)		SE/EE	3	Rota-rod/Morris/OFT	↑ Spatial memory ↓ cognitive deficits and anxiety phenotypes	20172507
(27)	*FMR1* gene knockout	ENR/STD	12	mother-infant interaction/T-maze/Situation freezing. etc	↓ ADHD in adulthood ↓ Heterosexual dependence ↓ Freezing behavior	25348604
(28)		SE/EE	8	OFT	↓ Internal exploration defects ↑ object habituation	16076950
(29)	*Oprm1* gene knockout	ENR/STD	4	USV/EPM/Social acceptance/Partner preference	↑ Social skills ↑ Social recognition in females	27274875
(30)	*Shank3* gene knockout	SE/UE	1.6	zero maze/stereotype. etc	No benefit, such as no improvement in self-grooming or orifice exploration ↑ Anxiety-like behavior	30317697
(31)	BTBR model	SE/EE	4.3	Grooming behavior/Repeated object exploration	↓ The carding time but keep the order unchanged	23813950
(32)	CNV-based model (Dp(11)17/+)	SE/EE	Unspecified	OFT/EPM/Rota-rod/FC	↓ Aggression and intensified repetitive exploration behavior ↓ Anxiety ↑ Balance to a lesser extent	22492990

↓: To reduce; ↑: To increase.

**Table 2:** Information on morphology and analysis of biological indicators.

Literature	Pattern making	Intervening measure	Intervention weeks	Molecular biological index	PMID
(16)	Exposure to VPA during pregnancy	SE/EE	16	↑ Microglia complexity ↑ Morphology and activity	33107086
(17)		SE/UE/PE	16	↑ Glutamic acid signaling protein content in somatosensory cortex, hippocampus and amygdala ↑ Corticosterone levels in plasma, amygdala, and hippocampus	26089770
(18)		SE/EE	3	↓ Loss of cerebellar Purkinje cells	
(19)		SE/EE	4.3	↑ Spontaneous firing of neurons in the barrel cortex	35738908
(21)		SE/EE/cluster needling	3	↑ Surviving frontal neurons ↑ IL-10 and ↓ IL-18 in frontal lobe	
(7)		SE/EE	4	↑ Synaptic density in CA1 ↑ BDNF-mRNA/PSD-95/Shank2/3	28655565
(22)	*Mecp2* gene mutation	SE/EE	2	↑ Syp, PSD95 and SGK-1	20634955
(23)		SH/EE	2	↑ BDNF expression in cortex, hippocampus, and cerebellum	18557922

↓: To reduce; ↑: To increase.

Given the specificity of the environmental enrichment intervention, with no uniform criteria for environmental conditions like intervention period, cage facilities, food type, and number of conspecifics, unfortunately, we were unable to produce a directional mean effect size estimate with 95% confidence intervals for intervention moderators. Although interventions cannot be quantified uniformly for comparison, the results in **Table 1** demonstrate the effectiveness of interventions on autistic symptoms. To explore the mechanisms behind the behavioral effects in depth, the literature provided thus far is sufficient. We will expand the literature by referring to the molecular biology indicators suggested in **Table 2**. We will also attempt to explain, as comprehensively as possible, the mechanisms behind EE and its impact on disease from the perspective of synaptic plasticity, neurogenesis, and molecular pathways.

### 3.3 Effects and underlying mechanisms of EE on ASD

#### 3.3.1 Effects of EE on social-deficit or ASD-like behaviors

Valproic acid (VPA), a non-genetic factor, plays a crucial role in mental disorders and mood conditions. Exposure to VPA significantly increases the risk of autism spectrum disorder (ASD) and is commonly used to develop a non-genetic ASD pathological model (12, 17, 33). Accumulating evidence suggests that exposure to an enriched environment (EE) ameliorates VPA-induced ASD pathological behaviors or symptoms (fear, anxiety, social withdrawal, and sensory abnormalities) by inducing neuroanatomical and behavioural changes (3, 10), including enhanced dendritic arborization, gliogenesis, neurogenesis, and improved learning (7, 12). In VPA-treated rats (12, 34, 35) and mice (7, 12), environmental enrichment significantly attenuated altered performance deficits (12), inhibited anxiety-like behavior, increased the number of social behaviors and social explorations, and improved social deficits and cognitive impairment, but showed no effect on hyperlocomotion (7, 12, 33). Further findings indicate that predictable environmental enrichment prevents the development of hyper-emotionality in the VPA-exposed group (17) and improves motor impairments (12), whereas unpredictable EE does not yield similar effects (17). Additionally, in the offspring of mice or *Rattus norvegicus* models exposed to VPA, environmental enrichment can improve autistic-like behavior symptoms through increased social interaction and repetitive movement (20, 36). Furthermore, continuous EE is reported to attenuate social behavior and cognitive function disturbances in the VPA-induced autistic-like model in a sex-dependent manner, at least in terms of behavioral performance (35, 37).

Over the past years, numerous genetic factors have been identified and confirmed to increase the risk of ASD (38, 39), and research on representative genes (Mecp2 and Shank3) mutant might elucidate more homogeneous subgroups within the spectrum (38, 40, 41). Mecp2 and Shank3 mutant model mice exhibit severe behavioural and neuropathological deficits associated with autistic spectrum developmental disorders (40, 42); restoring Mecp2 or Shank3 expression rescues Autism-like behaviors and social interaction deficits (40, 42). Interestingly, non-invasive and non-pharmacological EE intervention contributes to the improvement of neurological alterations caused by the Mecp2 or Shank3- transgenic models, preventing the development of motor discoordination and anxiety-related abnormalities (26, 43–45). On one hand, EE attenuates some neurological alterations, improving locomotor activity (46), increasing voluntary physical activity (26, 47), reducing motor coordination deficits (26, 33, 44, 48), and preventing the development of anxiety-related abnormalities (26, 47, 49, 50) and contextual fear conditioning in the several Mecp2 autistic models (39, 44). On the other hand, early environmental enrichment ameliorates behavioral abnormalities, increases anxiety‐like behavior in Shank3 Δe4–22 mice, and improves motor performance specifically in wild‐type mice (30, 51). Thus, different environmental factors provide significant benefits for the phenotypes resulting from RTT (Mecp2 or Shank3) related pathophysiologies (38–40).

Furthermore, based on the BTBR+Itpr3tf/J (BTBR) or fragile X mental retardation1 (FMR1) complex mutant, the genetically engineered animal has emerged as a well-validated model of autism, particularly in studies related to social deficits and repetitive behaviors (52–54). In both the BTBR-related genetic model (31, 37, 54, 55) and FMR1-knockout (FMR1-KO) mice (27, 28, 56), EE intervention significantly influences the quantity and quality of repetitive behaviors (27, 28, 31, 37, 54–56). Additionally, EE attenuates some neurological alterations in FXS mice, prevents the development of cognitive and anxiety-related abnormalities, and improves repetitive stereotyped behaviors (27, 28, 56). Interestingly, environmental stimulation may affect FMRP levels by activating glutamatergic signaling and FMRP-independent pathways in WT mice (28). Moreover, EE administration improves systemic metabolism, enhances learning and memory functions, inhibits behavioral phenotypes of anxiety, and lowers-order repetitive behaviors (27, 28, 55). However, it shows no effect on the overall quality of behaviors in the BTBR model, indicating that the improvements appear to occur in a dependent and sexual dimorphic manner (31, 37, 54, 55). Overall, the current data provides ample evidence supporting the effectiveness of EE intervention for neurodevelopmental mental disorders associated with specific genes (Mecp2, Shank3, and BTBR) linked to ASD syndrome. This strategy, at least, demonstrates notable plastic modulations of autism symptoms or associated neuro-alternations but appears to have limited influence on the alternation of these vital genes themselves.

#### 3.3.2 Improvement of EE on synaptic plasticity

EE can facilitate enhanced sensory perception, cognitive functions, and motor stimulation, thereby exerting beneficial effects on the regulation of neurobehavioral processes and psychotic behavior (3, 8, 10). The underlying mechanisms of EE on social deficits or ASD may involve the improvement of neuronal functions, neurogenesis, and synaptic plasticity (8, 57, 58). The main molecular pathways of EE contribute to structural changes in the brain and enhance synaptic plasticity (14). This is primarily implicated in learning, memory, and mood regulation in different hippocampal regions (such as CA1, CA3, and dentate gyrus), cortex (mPFC), or other regions.

Exposure of rodents to EE significantly improves performance in hippocampus-dependent learning and memory functions, regulates the balance of CA1 cellular excitability and synaptic plasticity (8, 58), and increases synaptic transmission in the dentate gyrus (DG) of the hippocampus (Hadi 58), particularly with short-term and/or periodic exposure to EE (8, 58). EE can improve hippocampal neurogenesis in the DG, decrease cellular apoptosis, myelination defects, microglial activation, and glutamatergic synaptic function in CA1 (8, 58, 59). Additionally, EE is found to induce brain plasticity via the spleen-brain connection network, where CD8+ T cell repartition of effector/central memory CD8+ T cells differs (58). Furthermore, animal research reveals that EE can regulate excitatory and, to a lesser extent, inhibitory synaptic density in the cerebellum and cortex, reverse cortical LTD deficits, and augment cortical neuroanatomical changes (BDNF) (26).

In addition to improving synaptic function, the beneficial effects of EE are closely associated with enhanced synaptic structure balance in basal dendritic length and spine density across various regions, including the cortex, hippocampus, striatum, and amygdala (7, 9, 60). On one hand, EE has demonstrated an ability to improve synaptogenesis and complex arbor formation, contributing to enhanced dendritic spines (7, 9, 61). On the other hand, EE significantly reverses decreased dendritic spine density, the number of mushroom spines, and disruptions in synapse remodeling (9, 13, 62–64). It also enriches the size distributions of presynaptic and postsynaptic spines and head sizes in hippocampal and cortex regions (62, 64). Additionally, EE can reduce abnormal ventricular volume (65), and enhance the expression of several synaptic marker genes critical to synaptic transmission and plasticity (44, 46). This seemingly demonstrates a brain region-specific effectiveness of physical EE, including an increase in hippocampus-dependent cognitive functions, improvement in Mpfc-associated anxiety, and a reduction in social interaction defects (11, 59, 66). Hence, EE improvements in synaptic plasticity are beneficial to early postnatal development and prevention of ASD phenotypes.

#### 3.3.3 Regulation of EE on vital molecular pathways

In addition to the prominent modulations of synaptic function, EE may play vital roles in managing autistic symptoms and pathology development through significant regulation of multiple targe-region proteins and signaling pathways that enhance neurological improvement in the brain. These pathways mainly involve neurogenesis and BDNF-related molecular pathways and pathological genes of ASD syndromes (8–11, 13, 14).

On one hand, EE treatment significantly increases BDNF levels in the cerebellum (48) and hippocampal regions (47) in Mecp2-related models. It improves the expression of synaptic proteins such as synaptophysin, syntaxin-1a, and synaptotagmin (22, 64), rescues the impaired BDNF-TrkB signaling in the prefrontal cortex and hippocampal regions in Fmr1 KO mice (56), and balances serum corticosterone via the hypothalamic–pituitary–adrenal (HPA) axis (47). EE can increase the gene expression of BDNF and its receptors in several brain areas, contributing to neural development and behaviors through the Ntrk2-TrkB and BDNF/TrkB-PLCγ1-CaMKII pathways (44, 46, 48, 55, 56, 67). Additionally, the transcriptional regulation of MeCP2 seems to target BDNF, exon 4, and Crh pathways (47), thus improving brain metabolic conditions and behavioral health (55). Thus, MeCP2 may be one of the main target genes involved in brain development, synaptic plasticity, and BDNF/TrkB pathways.

**Figure 2:**
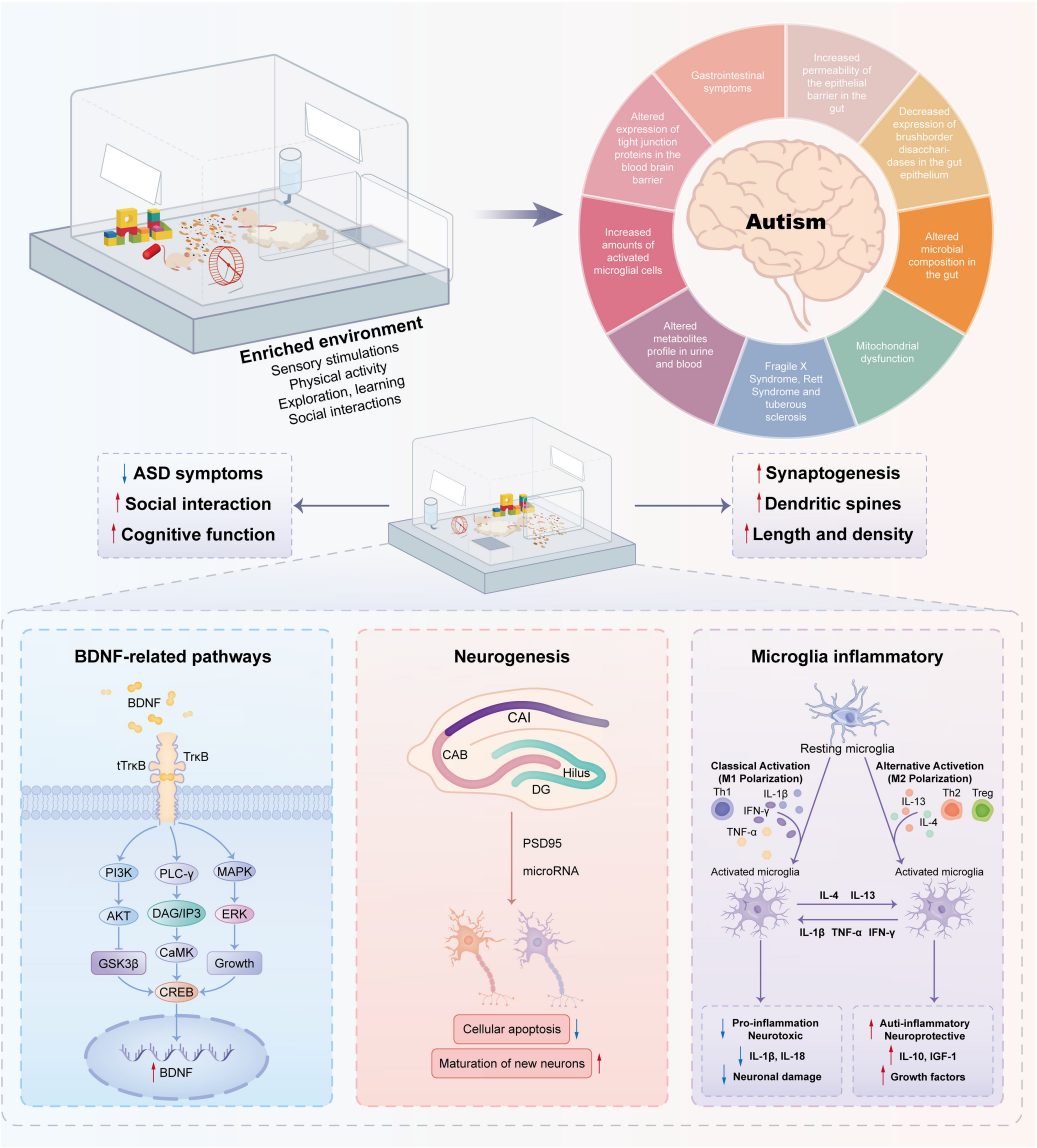
Molecular mechanisms associated with environmental enrichment to improve autism-like behaviors. Enriched environments have positive effects on autism spectrum disorders through complex molecular mechanisms. A pivotal element in this mechanism is Brain-Derived Neurotrophic Factor (BDNF), a neurotrophin essential for neuronal function. Stimulation from enriched environments boosts BDNF expression, promoting neurogenesis—the formation of new neurons crucial for cognitive processes. Moreover, these environments influence microglia, the brain’s immune cells, thereby inhibiting inflammation and creating a neuroprotective environment. The interplay among BDNF-related pathways, neurogenesis, and modulation of microglia contributes to the observed improvements in behavior.

On the other hand, EE can mitigate the loss of dendritic spines in the CA1 region, elevate the levels of postsynaptic density protein (PSD)-95 (26, 68), and SH3, and multiple ankyrin repeat domain 2 in the hippocampus (26). It also enhances the expression of several synaptic marker genes in autistic model animals (44, 46). Physical EE promotes neurogenesis in the DG (50), aids in the maturation and survival of new neurons (69), and influences microRNA expression, with upregulated miR-124 and miR-132 (**Figure 2**) (9, 50).

#### 3.3.4 Regulation of EE on microglia and inflammation

As a neurodevelopmental disorder, autism is associated with an abnormal increase in the number of microglia in several brain regions (70). Some studies have reported that the occurrence of autism is related to the pruning and stability of synapses (71, 72). Furthermore, others have demonstrated that microglia play a crucial role in the development of synapses by migrating to inflammatory sites and phagocytizing dead cells or their fragments (73, 74). These studies suggest that microglia contribute to autism-like behavior through synaptic pruning.

EE can ameliorate cognitive deficits and anxiety or depression-like symptoms by modulating microglial reactivity and functions in the brain (9, 15, 21, 36). It can reverse abnormal microglia numbers and volume in the DG (36) and enhance glial reactivities (15). Moreover, it inhibits the pro-inflammatory factor IL-18 and increases the level of the anti-inflammatory factor IL-10 (21).

In various animal models, EE has been shown to alter a range of inflammatory mechanisms, exerting anti-inflammatory or inhibitory effects on microglial “activation” (75). Some studies have indicated that EE can reduce inflammation and oxidative stress in the brain (76). For example, EE can decrease hippocampal IL-1β and serum monocyte chemoattractant protein-1, ultimately improving cognitive performance in aging rats (77). While microglia, as immune cells of the central nervous system, are closely related to EE, the special effects and mechanisms of EE on neuroinflammation and microglia-related functions are far from being explored and should be the focus of future studies.

The overall results reveal that EE exhibits significant regulation of molecular expression in different receptors in various brain regions, with receptor-dependent and sexual dimorphic manners (17, 37, 55). For instance, EE notably upregulates the levels of Glu2B in the S1 cortex, CaMKII in the dorsal hippocampus, and GluN2B in the amygdaloid nucleus but decreases the levels of GluN1 in the ventral hippocampus (17). EE affects the expression of NMDAR1s and CB1Rs in the cerebellar cortex in BTBR model mice (55). However, it seems that EE demonstrates a differential regulation in the expression of different synaptic receptors between males and females (35, 37, 55). Continuous EE can alleviate cognitive dysfunction in autistic rats, at least at the behavioral level, and its impact depends on gender. Females performed better than males in discrimination tasks and acoustic startle reflexes. In contrast, males were better than females in the three‐chamber social interaction test (19).

In general, EE stimulation exerts beneficial effects via BDNF-related pathways, neurogenesis, and microglia-related inflammation, as shown in **Figure 2**. Thus, it can be optimized as a treatment option with the use of nonpharmacological interventions for the treatment of social defect disorders in ASD.

## 4 Discussion

EE interventions aim to provide individuals with a stimulating and engaging environment, fostering enhancement in their physical, cognitive, and social abilities. This approach has demonstrated benefits for individuals with various mental illnesses, including depression, schizophrenia, and bipolar disorder. For example, in a randomized trial investigating EE in schizophrenia, patients exposed to EE exhibited improved cognitive performance and milder symptoms (78, 79).

In the field of neuroscience, EE is increasingly recognized for its potential to enhance synaptic plasticity. Exposure to EE induces an upregulation in BDNF expression and TrkB activation, thereby promoting the formation and maintenance of new synaptic connections (6). Additionally, EE enhances synaptic plasticity by increasing the formation of new neurons in the hippocampus (12). Advanced imaging techniques such as magnetic resonance imaging (MRI) or positron emission tomography (PET) scans demonstrate that EEs enhance protein recombination and activate brain glucose metabolism, thereby contributing to brain remodeling (80).

These studies collectively illustrate that exposure to EE results in increased activity in brain regions associated with learning and memory, leading to improved cognitive and behavioral performance (26). Compared with previous studies, our work delves deeper into the mechanisms underlying various behavioral phenotypic variations induced by enriched environments, exploring gene phenotypes, molecular signaling pathways, synaptic plasticity, and microglia-associated inflammation. Our ongoing research aims to unveil molecular and loop-level insights. Through this systematic review, we hope to provide a clear direction for autism-related research.

In animal model research, enriched sensorimotor environments enable rodents to overcome a spectrum of neurological challenges, including those induced in animal models of autism. Some scholars posit that environmental enrichment could serve as an effective method for treating a wide range of symptoms in human autism patients. In a randomized controlled trial, children with autism were divided into a sensorimotor enrichment group and a standard care control group, with long-term observation conducted for 6 months. The findings revealed significant improvement in children with autism in the sensorimotor enrichment group compared to the control group (81, 82). Moreover, personalized treatment guidance on the Internet has led to substantial improvements in the condition of children with autism who adhere to their parents’ arrangements (83). These studies suggest that environmental enrichment appears effective in improving certain symptoms of autism.

However, further research is needed to comprehensively understand the mechanisms underlying these effects and determine the most effective ways to implement EE interventions in individuals with autism and other neurodevelopmental disorders.

## Data availability statement

The original contributions presented in the study are included in the article/supplementary material. Further inquiries can be directed to the corresponding author.

## Author contributions

YL: Conceptualization, Investigation, Methodology, Writing – original draft, Writing – review & editing. JL: Formal analysis, Writing – review & editing. JZ: Formal analysis, Writing – review & editing. WG: Writing – review & editing. WX: Writing – review & editing.
