# Supplementary material for: Molecular insights into enriched environments and behavioral improvements in autism: a systematic review and meta-analysis
Source: Front Psychiatry. 2024 Feb 1;15:1328240. doi: 10.3389/fpsyt.2024.1328240 (PMC10867156; doi:10.3389/fpsyt.2024.1328240)
Supplement: Supplementary file 1 [file Presentation_1.pdf]

Supplementary Material 1: Electronic searchers

MEDLINE (July 25, 2022)

((("Autistic Disorder"[Mesh]) OR ((((((((((Disorder, Autistic[Title/Abstract]) OR (Disorders, Autistic[Title/Abstract])) OR (Kanner's Syndrome[Title/Abstract])) OR (Kanner Syndrome[Title/Abstract])) OR (Kanners Syndrome[Title/Abstract])) OR (Autism, Infantile[Title/Abstract])) OR (Infantile Autism[Title/Abstract])) OR (Autism[Title/Abstract])) OR (Autism, Early Infantile[Title/Abstract])) OR (Early Infantile Autism[Title/Abstract])) OR (Infantile Autism, Early[Title/Abstract]))) AND ((((Environmental enrichment[Title/Abstract]) OR (enriched environment[Title/Abstract])) OR (environmental richness[Title/Abstract])) OR (enrichment environment[Title/Abstract]))) AND (("Animal Experimentation"[Mesh]) OR ((((((((((Experimentation, Animal) OR (Animal Research)) OR (Research, Animal)) OR (Animal Experimental Use)) OR (Animal Experimental Uses)) OR (Experimental Use, Animal)) OR (Experimental Uses, Animal)) OR (Animal Experiments)) OR (Animal Experiment)) OR (Experiment, Animal)) OR (Experiments, Animal)))

EMBASE (July 25, 2022)

[experimentation, AND animal OR (animal AND research) OR (research, AND animal) OR (animal AND experimental AND use) OR (animal AND experimental AND uses) OR (experimental AND use, AND animal) OR (experimental AND uses, AND animal) OR (animal AND experiments) OR (animal AND experiment) OR (experiment, AND animal) OR (experiments, AND animal) OR 'animal experiment'/exp] AND ['environmental enrichment'/exp OR 'environmental enrichment':ab,ti OR 'enriched environment':ab,ti OR 'environmental richness':ab,ti OR 'enrichment environment':ab,ti] AND ['disorder, autistic':ab,ti OR 'disorders, autistic':ab,ti OR 'kanner syndrome':ab,ti OR 'kanners syndrome':ab,ti OR 'autism, infantile':ab,ti OR 'infantile autism':ab,ti OR autism:ab,ti OR 'autism, early infantile':ab,ti OR 'early infantile autism':ab,ti OR 'infantile autism, early':ab,ti OR 'autism'/exp]

ISI (July 25, 2022)

TS= (Autistic Disorder OR Disorder, Autistic OR Disorders, Autistic OR Kanner's Syndrome OR Kanner Syndrome OR Kanners Syndrome OR Autism, Infantile OR Infantile Autism OR Autism OR Autism, Early Infantile OR Early Infantile Autism OR Infantile Autism, Early)
TS= (Environmental enrichment OR enriched environment OR environmental richness OR enrichment environment)
TS= (Animal experiment OR Experimentation, Animal OR Animal Research OR Research, Animal OR Animal Experimental Use OR Animal Experimental Uses OR Experimental Use, Animal OR Experimental Uses, Animal OR Animal Experiments OR Animal Experiment OR Experiment, Animal OR Experiments, Animal)

Other search forms do not support export search, and we can provide screenshots of the day as proof of search if needed.
